# CADD-ThresholdApp: an interactive web application for calibrating CADD scores using ClinVar

**DOI:** 10.1186/s12859-026-06675-x

**Published:** 2026-09-24

**Authors:** Cora Leifheit, Martin Kircher, Max Schubach

**Affiliations:** 1 Berlin Institute of Health at Charité – Universitätsmedizin Berlin, Luisenstraße 65, 10117 Berlin, Germany; 2 Institute of Human Genetics, University of Luebeck, University Hospital Schleswig-Holstein, Campus Luebeck, Ratzeburger Allee 160, 23562 Lübeck, Germany

**Keywords:** Variant prioritization, CADD score, ClinVar, Gene panels, Visualization, Rare diseases, Web application

## Abstract

**Background:**

The Combined Annotation Dependent Depletion (CADD) score is one of the most widely used genome-wide variant deleteriousness scores. In practice, however, the distribution of CADD scores differs across genes and gene sets and may also vary between genome builds and CADD releases, complicating the choice of a single threshold for prioritizing potentially pathogenic variants in routine diagnostics.

**Results:**

We present CADD-ThresholdApp, an interactive web application that supports threshold selection by visualizing CADD-score distributions of clinically classified variants from ClinVar and by reporting threshold-dependent performance metrics. The app enables comparisons across genome releases (GRCh37/GRCh38) and CADD versions (v1.6/v1.7), supports user-defined genes and gene lists, as well as curated gene panels from PanelApp. CADD-ThresholdApp is implemented in a modular, extensible framework and can be adapted to additional variant resources and deleteriousness scores. The app is available as a hosted web application at https://cadd-threshold.kircherlab.bihealth.org/ and can be installed locally via container, PyPI, Bioconda, or from source.

**Conclusions:**

Overall, CADD-ThresholdApp provides a practical and transparent framework for context-specific threshold calibration to support more informed variant interpretation in research and diagnostics.

## Background

Human genome sequencing has become a standard practice in genetic diagnostics [1]. Sequencing an individual genome identifies many variants, making it essential to distinguish potentially causal (pathogenic) from neutral variants during clinical interpretation. To support variant prioritization, tools such as Combined Annotation Dependent Depletion (CADD) [2] are widely used. CADD assigns deleteriousness scores using a linear model and provides PHRED-like scaled scores to enable comparison across approximately 9 billion potential single nucleotide variants. Universal CADD cutoffs for including or excluding candidate variants are generally not recommended, because optimal thresholds depend on factors such as gene, variant consequence, and study design [3]. In practice, however, thresholds are widely used [4], and scores may be recalibrated [5, 6] or retrained [7] for specific (clinically relevant) gene sets.

Existing resources address related but distinct needs. Tools such as VEPerform [8] allow gene-level evaluation of variant effect predictors using precision–recall-based summary statistics, but focus on comparing predictors for a fixed evidence threshold rather than allowing interactive exploration of user-defined cutoffs across the full score range. Decision-support systems such as the ClinGen Pathogenicity Calculator, InterVar, or CardioClassifier [8–10] operationalize ACMG/AMP evidence codes [11] and treat computational scores as one fixed evidentiary input among several, rather than exposing the underlying score distributions and threshold trade-offs to the user. Recalibration and retraining studies typically report a single optimized threshold or model for a specific gene or region [5–7], rather than providing an interactive tool that lets users choose and immediately evaluate their own thresholds.

We present CADD-ThresholdApp, an interactive web application that enables users to explore how clinically classified variants are distributed across CADD PHRED scores and to evaluate threshold-dependent performance metrics. CADD-ThresholdApp differs from all of the resources above by providing an interactive and directly comparable threshold-calibration framework across genome builds, CADD releases, and user-defined gene subsets, without requiring a fixed evidentiary framework or model retraining. The data underlying the app were generated from Clinical Variation (ClinVar) database variants [12] scored with CADD. The app allows users to (i) explore precomputed metrics across the full range of PHRED scores within classified ClinVar variants, (ii) compare genome releases (GRCh37 and GRCh38) and CADD versions (v1.6 and v1.7), and (iii) compute metrics for selected genes and gene panels, including predefined panels from PanelApp [13]. By visualizing pathogenic and benign variant distributions across thresholds, the tool helps researchers and clinicians select cutoffs tailored to their analyses, including explicit consideration of false-negative rates.

## Implementation

### Data acquisition

Variants with known clinical classifications were retrieved from ClinVar [12] (download date April 22, 2026). To focus on high-quality and reliable entries, variants were filtered by review status, retaining only those with at least two stars (“criteria provided, multiple submitters, no conflicts”, “reviewed by expert panel”, or “practice guideline”). Variants were further restricted to those with clinical significance containing “benign”, “likely benign”, “pathogenic”, or “likely pathogenic”, and were then stratified by genome release (GRCh37 and GRCh38). For visualization by clinical significance, entries containing both “benign” and “likely benign” or both “pathogenic” and “likely pathogenic” were assigned to “likely benign” or “likely pathogenic”, respectively. For metric calculation, clinical significance was binarized into two classes: positive = {“pathogenic”, “likely pathogenic”} and negative = {“benign”, “likely benign”}.

Filtered variants were scored using CADD v1.6 and v1.7 via the CADD website [14]. PHRED-scaled scores as well as CADD annotations were retained and merged with corresponding ClinVar annotations to create a unified table containing clinical classifications, CADD PHRED scores, and variant metadata.

**Fig. 1:**
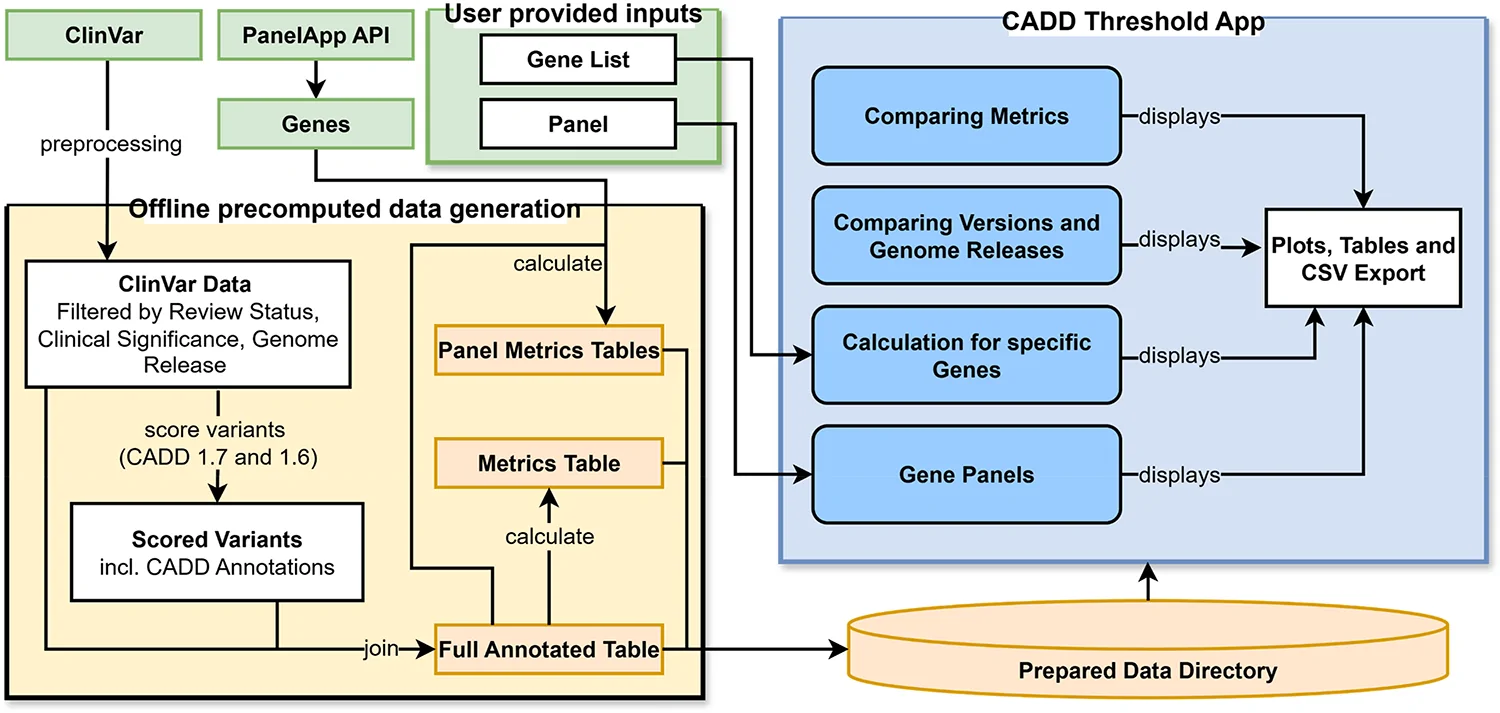
Overview of the processing pipeline and the CADD-Threshold App. ClinVar and PanelApp data are collected and curated to derive summary metrics and variant annotations from ClinVar and CADD. Within the app, users can explore precomputed analyses (global threshold and version/genome comparisons) or conduct custom analyses using user-defined gene lists or selected panels. Users may also optionally supply a directory containing a prepared custom dataset, which replaces the packaged data source. All analysis paths result in interactive visualizations, tables, exportable summaries, and the support indicator

To summarize threshold-dependent performance, multiple metrics were calculated across PHRED cutoffs by comparing CADD PHRED scores with the binarized ClinVar labels. These metrics included true negatives, false positives, false negatives, true positives, precision, recall, F1 score, F2 score, support, accuracy, balanced accuracy, false positive rate, and specificity. For each threshold T, variants with CADD PHRED ≥ T were classified as positive predictions and variants with CADD PHRED < T as negative predictions. The full workflow, implemented in Snakemake [15], is documented in the accompanying GitHub repository [16], enabling users to reproduce the analysis and, if desired, process their own datasets. Figure 1 provides an overview of the data acquisition process and its integration with the CADD-Threshold App.

### Customized data

To reproduce our results or analyze user-provided datasets, we provide the above-mentioned Snakemake workflow. As input, users supply a variant table containing chromosome (CHROM), position (POS), reference allele (REF), and alternate allele (ALT). For performance evaluation, the table must also contain a clinical significance column encoded as a binary class representing positive and negative variants, for example benign and pathogenic.

In the first step, the preprocessing workflow sorts variants by chromosome and position and formats them for CADD scoring, splitting the dataset into multiple Variant Call Format (VCF) files of up to 100,000 variants each, which is the maximum number of variants supported per CADD website upload. If additional filtering is required beforehand, for example restricting ClinVar data to two-star entries [12], users can perform this step using the recommended commands provided in the README. In general, the input file is expected to already be filtered according to the intended use case.

The resulting VCF files can then be uploaded to the CADD websites ([14] or [17]) or scored locally using CADD-scripts [18] to obtain PHRED-scaled scores. The score files are then downloaded and stored in the workflow project directory. In the second step, running the all_metrics workflow merges the CADD-scored output with the original variant table and computes the evaluation metrics by comparing CADD PHRED scores with the provided clinical significance labels. These metrics, together with the associated variant distributions, can then be displayed in CADD-ThresholdApp using the combined table from CADD and the input dataset (e.g., ClinVar).

### Web application

The CADD-ThresholdApp is implemented using Shiny for Python and can be accessed via our website [19] or installed locally via container, PyPI, Bioconda, or from source [20]. When the project is installed as a package, the underlying data must be downloaded separately [21], and the corresponding data path must be specified during start (cadd-threshold-app --data /path/to/data). The data are versioned separately from the source code to decouple updates of ClinVar and CADD versions from the core panel app and to provide transparency.

**Fig. 2:**
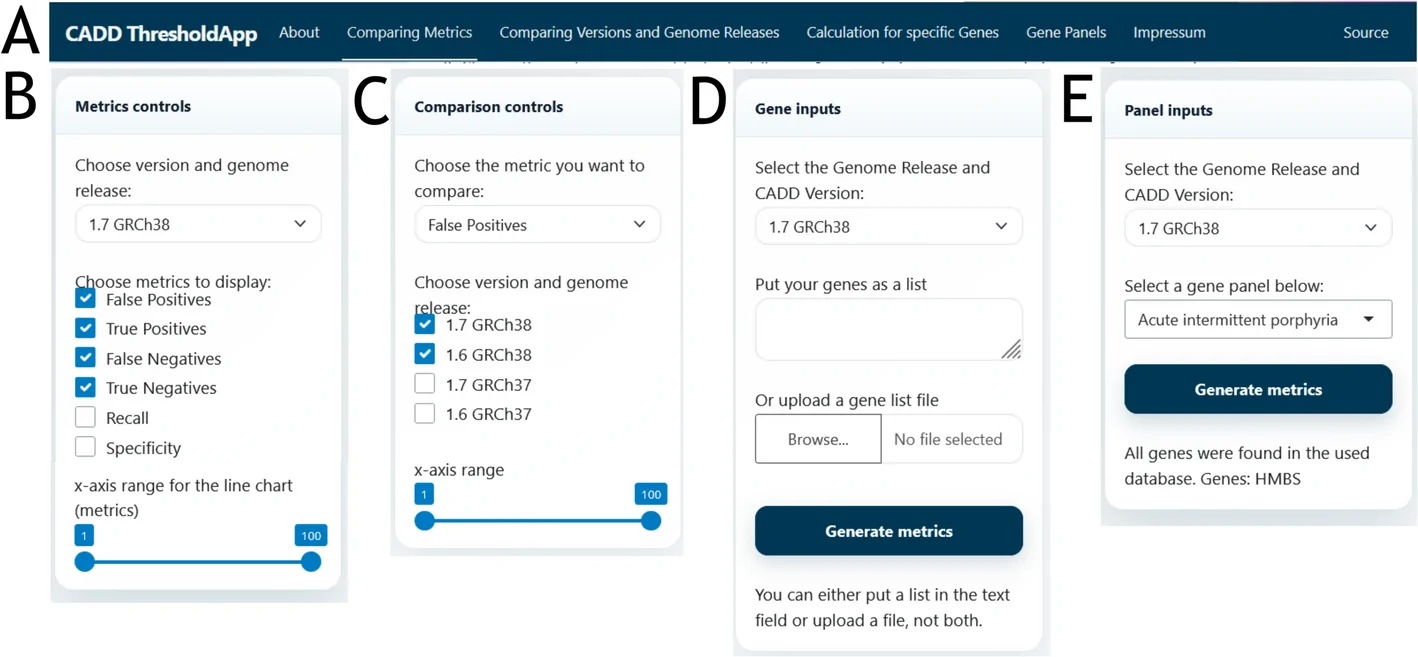
Overview of the web application functionalities. **A** Navigation bar linking to all app functionalities. **B** Comparing Metrics tab, where users can select a genome release and CADD version, choose performance metrics to display and define the threshold range for visualization. **C** Comparing Versions and Genome Releases tab, where users can select different genome releases and CADD versions to compare with a selected metric. **D** Calculation for specific Genes tab, where users can enter or upload a list of genes and choose a genome release and CADD version to compute gene-specific threshold-dependent metrics when pressing the ‘Generate Metrics’ button. **E** Gene Panels tab, where users can choose a gene panel (from PanelApp) and choose a genome release and CADD version to compute panel-specific threshold-dependent metrics when pressing the ‘Generate Metrics’ button

The CADD-ThresholdApp web interface is organized into tabs corresponding to three main functionalities (see Fig. 2):

#### Exploring metrics across thresholds

**Fig. 3:**
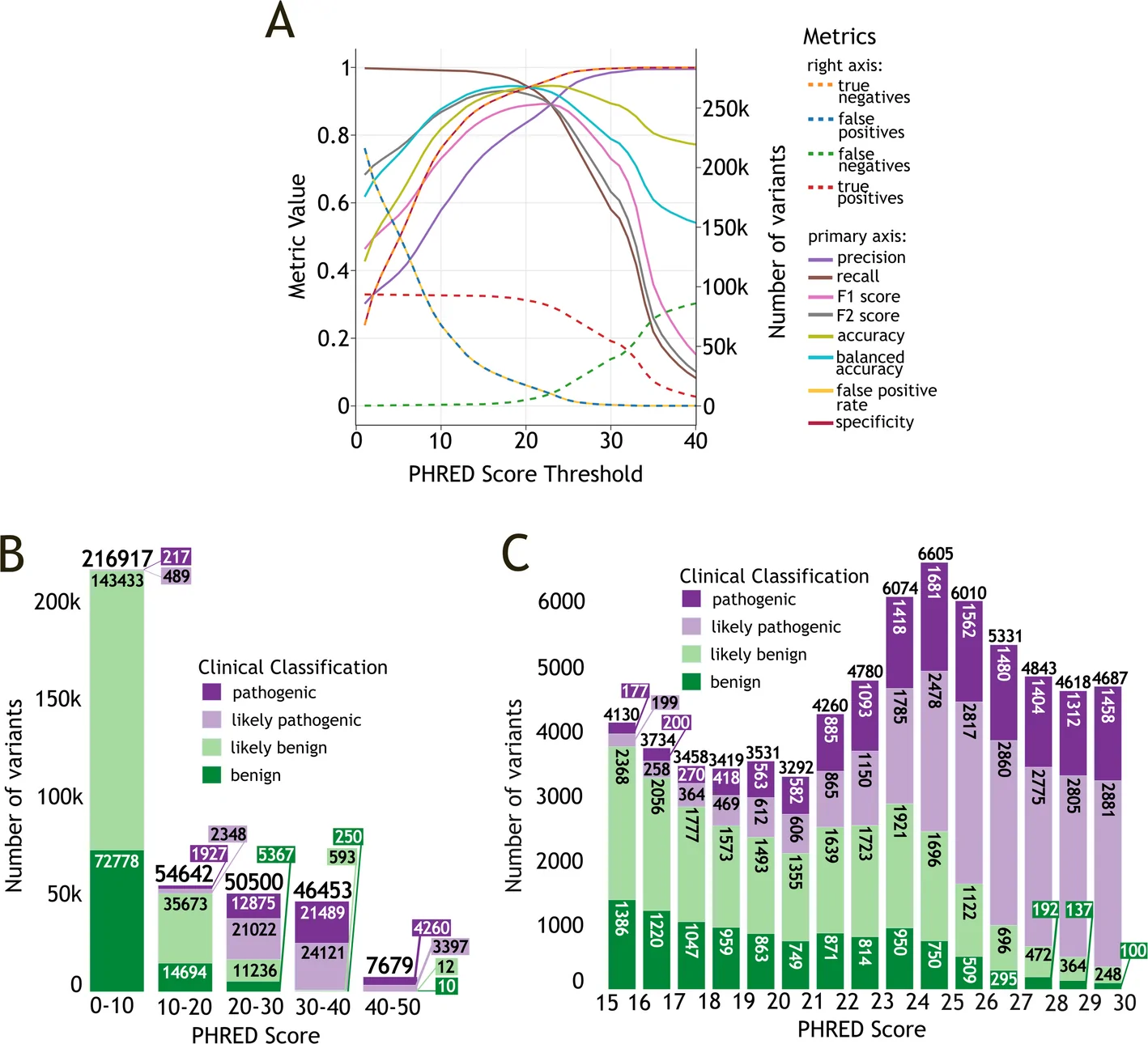
Variant distribution and performance metrics across CADD PHRED score thresholds in CADD-ThresholdApp. Panels show ClinVar variants downloaded on 22 April 2026, restricted to entries with review status ≥ two stars and clinical significance in {benign, likely benign, pathogenic, likely pathogenic} after binarization into positive and negative classes. **A** Threshold-dependent metrics (true negatives, false positives, false negatives, true positives, recall, specificity, false positive rate, accuracy, balanced accuracy, F1-score, F2-score and precision) for GRCh38, CADD v1.7, from PHRED 0 to 40. **B **Distribution of ClinVar variants for GRCh38, CADD v1.7, from PHRED 0 to 40 in steps of 10. **C** Distribution of ClinVar variants for GRCh38, CADD v1.7, from PHRED 0 to 40 in steps of 1

Precomputed performance metrics are displayed across CADD PHRED thresholds from 0 to 100, allowing users to interactively inspect how each metric changes by increasing the threshold (see Fig. 3A). Users can select which metrics to display and adjust the threshold range. False negatives, false positives, true negatives, and true positives are shown as absolute values, whereas the remaining metrics are displayed as relative values. In addition, the app shows variant distributions across thresholds in steps of ten (see Fig. 3B) and one (see Fig. 3C), as well as the distribution of pathogenic variants across thresholds together with their consequences from the CADD annotations (e.g., missense, stop-gain, synonymous). For the step size of one, the threshold range can be adjusted for detailed inspection.

#### Comparing genome releases and CADD versions

**Fig. 4:**
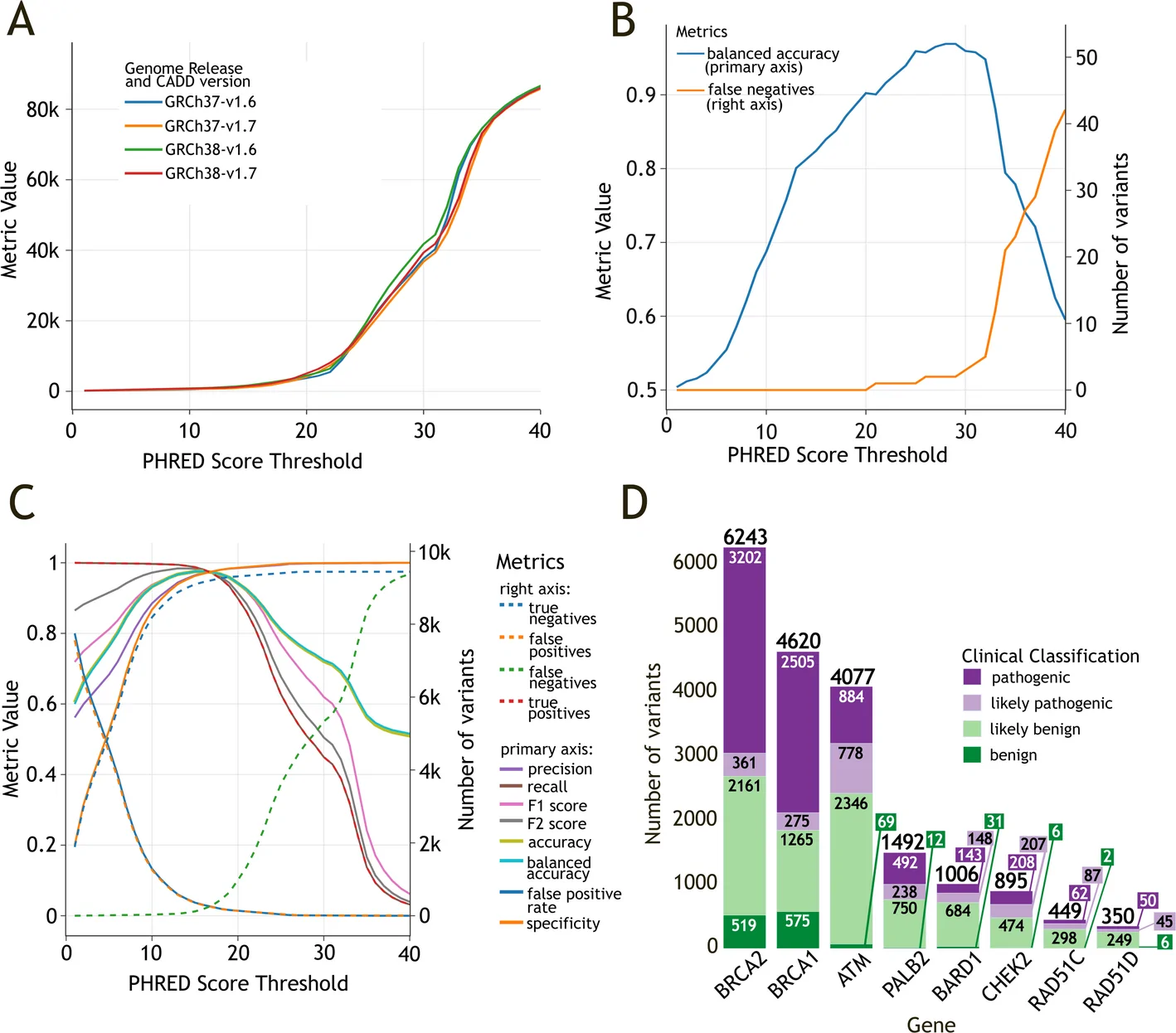
Variant distribution and performance metrics across CADD PHRED score thresholds in CADD-ThresholdApp. Panel A shows all retrieved variants from ClinVar (see underlying data). Panel B shows only ClinVar variants of the gene ZEB2. Lower panels (C and D) show only ClinVar variants of genes within the panel “Inherited breast cancer and ovarian cancer” taken from the PanelApp. **A** Comparison of false negatives for GRCh37 and GRCh38 in combination with CADD v1.6 and CADD v1.7, from PHRED 0 to 40. **B **Threshold-dependent metrics (false negatives and balanced accuracy) for GRCh38, CADD v1.7, from PHRED 0 to 40 of ClinVar variants within a gene subset (*n* = 1). **C** Threshold-dependent metrics, CADD v1.7-GRCh38, from PHRED 0 to 40, of ClinVar variants within a gene subset (*n* = 8). **D **Number of variants per gene in the panel “Inherited breast cancer and ovarian cancer”, stratified into “benign”, “likely benign”, “pathogenic”, and “likely pathogenic” by clinical significance (CADD v1.7-GRCh38)

Users can compare results between the human genome reference releases GRCh37 and GRCh38, as well as between different CADD versions (v1.6 and v1.7), using selected metrics (see Fig. 4A). This enables assessment of how genome release or model version affects threshold behavior.

#### Computing gene- and panel-specific metrics

Users can restrict analyses to variants associated with selected genes based on CADD gene annotations, and the app recomputes threshold-dependent metrics on this subset (see Fig. 4B). The resulting metrics are displayed together with a summary table listing the selected genes and additional metadata, which can be downloaded for further inspection. For each gene, the app also reports the number of variants available in the database, stratified into “benign”, “likely benign”, “pathogenic”, and “likely pathogenic”.

Similarly, users can analyze predefined gene panels from PanelApp (accessed May 4th, 2026). For a selected panel, the app indicates which genes are present in the database and which are missing and then provides the same metric summaries as in the gene-based workflow (see Fig. 4C and D). These functions help users understand threshold distributions for the specific gene subsets used in their analyses and support more tailored threshold selection for specific diseases.

### Statistical robustness of gene- and panel-specific metrics

To assess the stability of gene- and panel-specific analyses at low variant counts, we performed a bootstrap subsampling analysis using genes with large numbers of ClinVar variants. Pathogenic and benign variants were analyzed separately for each genome build (GRCh37 and GRCh38) and CADD version (v1.6 and v1.7). For each stratum, we selected genes with at least 200 variants and treated the corresponding gene-specific CADD PHRED score distributions as reference distributions. If more than 100 genes met this criterion in a given stratum, we randomly selected 100 eligible genes for analysis; otherwise, all eligible genes were analyzed.

For each gene, we repeatedly generated smaller subsamples across a range of sample sizes, estimated bootstrap confidence intervals for the mean and standard deviation of the CADD PHRED scores, and quantified, for each subsample size *n*, the fraction of genes for which the bootstrap confidence intervals for both mean and standard deviation contained the corresponding full gene-specific values in at least 90% of repeated subsamples. From these aggregated coverage curves, we defined sample-size thresholds *n*_25_, *n*_50_, and *n*_75_ as the smallest sample sizes at which at least 25%, 50%, and 75% of eligible genes, respectively, met this 0.90 coverage criterion. These thresholds are used to classify sample sizes underlying gene- and panel-specific metrics into “low”, “moderate”, or “good” support categories.

## Results

The initial ClinVar dataset comprised 8,920,416 variant entries. After filtering, 758,130 entries remained, of which 379,015 mapped to GRCh37, 378,983 to GRCh38, and 132 were discarded because the genome release could not be unambiguously determined. After scoring the variants with CADD v1.6, 377,966 variants were left for GRCh37 and 377,835 variants for GRCh38. Scoring the variants with CADD v1.7, left 377,965 variants for GRCh37 and 377,834 variants for GRCh38. This included variants from 11,793 to 13,018 genes (GRCh37 CADD v1.6: 12,844; GRCh37 CADD v1.7: 12,863; GRCh38 CADD v1.6: 13,018; GRCh38 CADD v1.7: 11,793). For the differences in the number of genes, please note that CADD GRCh37 releases use Ensembl release 75, whereas GRCh38 CADD v1.6 uses Ensembl release 95 and CADD v1.7 uses Ensembl release 110, respectively.

CADD-ThresholdApp supports four main types of use-cases. In most examples shown here, we used GRCh38 together with CADD v1.7 for better comparison.

First, the app allows inspection of the full dataset to evaluate how the number of false negatives varies across CADD PHRED thresholds. We prioritize false negatives to minimize the risk of missing potentially deleterious variants. In our dataset, the number of false negatives remains relatively stable up to a threshold of 20 (from 227 to 4,994 variants) but then increases rapidly between thresholds 20 and 40 (from 6,339 to 86,023 variants) after which they begin to plateau (see Fig. 3A). This pattern illustrates how CADD-ThresholdApp can be used to identify a reasonable general threshold (around 20 in our dataset) and, importantly, confirms that the app reproduces previously reported behavior of CADD scores in ClinVar-derived data [22]. Consistent with this observation, the distribution plots show that the number of pathogenic variants also increases from a threshold of around 20 onward (see Fig. 3B and C).

Second, the app allows comparison of different genome releases and CADD versions. We compared false negatives for GRCh37 and GRCh38 in combination with CADD v1.6 and v1.7. Overall, the results were very similar (see Fig. 4A). These comparisons demonstrate that CADD-ThresholdApp recapitulates the expected similarity of CADD performance across GRCh37 and GRCh38. In this sense, the genome-build and version comparisons serve as validation examples for the app rather than novel findings about CADD itself. Furthermore, the latest CADD version (v1.7) does not show an improved performance compared to version 1.6 when using this specific ClinVar dataset as benchmark. However, the CADD authors were able to show that there are indeed differences between the versions when using other datasets [2].

Third, the app can be used for gene-specific analyses, which are particularly relevant when only variants in selected genes are of interest. In such cases, threshold behavior observed in the full dataset may not accurately reflect the characteristics of the subset used in the specific use-case. As an example, we examined ZEB2, a gene associated with Mowat-Wilson syndrome. For this gene, pathogenic and benign classes are imbalanced (52 vs. 128), therefore the balanced accuracy helps to interpret the gene better. Balanced accuracy was highest at thresholds 28 and 29 (0.97), with only 2 false negatives (see Fig. 4B). This suggests that for ZEB2 a higher threshold may be appropriate than the one chosen based on the full dataset.

Fourth, to make it easier to analyze and view already established and generally larger gene sets associated with specific diseases, users can choose panels from PanelApp and view the corresponding metrics. Here, we analyze the panel “Inherited breast cancer and ovarian cancer”. The panel contains 8 genes (BRCA1, BRCA2, ATM, PALB2, BARD1, CHEK2, RAD51C, and RAD51D) with 19,132 variants in total (see Fig. 4D). This bar chart clearly indicates that the three genes BRCA2, BRCA1, and ATM contribute to most of the variants. Therefore, the other five genes are underrepresented in all metrics (see Fig. 4C). If necessary, the user can recompute the metrics based on a subset of the panel for comparison. For this specific panel false negatives increase after a threshold of 15, which differs from the overall ClinVar dataset where we see the increase from threshold 20 onwards (compare with Fig. 3A). This suggests that a lower threshold might be suitable for breast and ovarian cancer diagnostics.

The resulting bootstrap-derived thresholds for each genome build, CADD version, and variant class are summarized in Table 1. In general, the bootstrap analysis suggested similar thresholds across pathogenic and benign variants, genome releases, and CADD versions. with $\:{n}_{25}$ typically around 30–50, $\:{n}_{50}$around 40–60, and $\:{n}_{75}$around 70–90 variants.

**Table 1:** Bootstrap-derived support thresholds for gene-level CADD PHRED summaries across genome builds, CADD versions, and variant classes

Variant class	Genome build	CADD version	Eligible genes	$\:{n}_{25}$	$\:{n}_{50}$	$\:{n}_{75}$
Pathogenic	GRCh37	v1.6	69	50	50	70
Pathogenic	GRCh37	v1.7	69	40	50	80
Pathogenic	GRCh38	v1.6	68	30	50	70
Pathogenic	GRCh38	v1.7	69	40	60	80
Benign	GRCh37	v1.6	201	30	40	70
Benign	GRCh37	v1.7	205	30	50	80
Benign	GRCh38	v1.6	200	30	50	90
Benign	GRCh38	v1.7	201	30	50	70

For each combination of genome build (GRCh37/GRCh38), CADD version (v1.6/v1.7), and variant class (pathogenic/benign), we selected genes with at least 200 variants and performed a bootstrap subsampling analysis to quantify the stability of gene-specific CADD PHRED score distributions. When more than 100 genes satisfied this minimum-count criterion, a random subset of 100 eligible genes was analyzed; otherwise, all eligible genes were included. Thresholds $\:{n}_{25}$, $\:{n}_{50}$, and $\:{n}_{75}$ denote the smallest subsample sizes at which at least 25%, 50%, and 75% of eligible genes, respectively, achieved a coverage rate of at least 0.90 for both the mean and standard deviation relative to the corresponding full gene-specific distribution. These thresholds are used in CADD-ThresholdApp to classify the sample sizes underlying gene- and panel-specific metrics into “Low support”, “Moderate support”, and “Good support” categories

## Discussion

With the described functionality, the CADD-ThresholdApp supports selection and justification of CADD PHRED cutoffs by visualizing score distributions and reporting threshold-dependent performance metrics, including explicit false-negative behavior, for the full dataset as well as gene- and panel-restricted subsets. This enables comparison of threshold behavior across GRCh37/GRCh38 and CADD releases and supports workflow- or disease-specific cutoff selection.

The tool is intended to support variant filtering and prioritization rather than formal clinical classification. In a typical diagnostic workflow, clinicians or analysts first generate a list of candidate variants (for example by restricting to genes of interest, variant types, and allele frequency thresholds) and then apply deleteriousness scores such as CADD to decide which variants merit closer inspection. Our tool fits into this stage by helping users select context-appropriate CADD PHRED cutoffs for specific genes or gene panels, benchmarking how different thresholds affect false-negative and false-positive behavior in their setting. The resulting recommendations can be used to refine candidate variant lists before applying formal classification frameworks (e.g., ACMG/AMP guidelines), which remain the primary basis for clinical interpretation and reporting. In this way, CADD-ThresholdApp supports the filtering and prioritization step without replacing or modifying the downstream clinical classification process.

As the default underlying data are based on ClinVar, the observed threshold-dependent performance reflects the composition of ClinVar and may be influenced by gene- and disease-specific ascertainment, as well as by the subset of variants with curated qualitative clinical classifications [23]. A related limitation is the potential circularity that can arise when ClinVar is used both to train and to evaluate predictor behavior. However, overlap between ClinVar variants and the CADD training data is negligible. Still, because clinically observed variants may be enriched for deleteriousness, thresholds derived from ClinVar should be interpreted cautiously. Computational predictors are considered supporting evidence under ACMG/AMP guidelines, and expert curation remains essential [11]. In the future, deep mutational scanning may help overcome this limitation [24], although genome-wide or panel-wide benchmarking is currently not feasible due to the limited number of assayed genes. Nevertheless, such datasets are valuable for benchmarking predictors themselves, as demonstrated previously for CADD [2]. To support alternative use cases, we provide a Snakemake workflow that enables users to generate app-compatible datasets from custom variant tables using the same preprocessing and metric-computation steps.

For future extensions, we plan to add support for additional variant effect predictors and variant resources, enable side-by-side threshold comparison to highlight where predictors might disagree, and expand stratified analyses to make threshold selection more interpretable and to better reflect the heterogeneity of variant effects across biological settings.

## Conclusions

CADD-ThresholdApp combines curated clinical labels from ClinVar with harmonized CADD scores and precomputed threshold-dependent performance metrics in an interactive and reusable web resource. By enabling comparisons across genome releases, CADD versions, and selected gene sets or gene panels, the app provides a transparent framework for context-specific calibration of CADD PHRED cutoffs. In this way, CADD-ThresholdApp supports more informed and reproducible variant prioritization in both research and diagnostic settings. The modular design further provides a foundation for future extension to additional variant resources and deleteriousness scores.

## Data Availability

The datasets generated and analyzed during the current study and supporting the conclusions of this article are available in the data for cadd-threshold-app repository, [https://doi.org/10.5281/zenodo.19204078]. The datasets analyzed in our study were originally obtained from [https://ftp.ncbi.nlm.nih.gov/pub/clinvar/tab_delimited/] under variant_summary.txt.gz. CADD-ThresholdApp is available as a hosted web application at [https://cadd-threshold.kircherlab.bihealth.org/]. Source code is openly available in the cadd-threshold repository, [https://github.com/kircherlab/CADD_threshold_app], with data-processing workflows in the CADD_threshold_analysis repository,[https://github.com/kircherlab/CADD_threshold_analysis] and archived resources at the kircherlab/CADD_threshold_app repository, [https://doi.org/10.5281/zenodo.19565035]. The app can be installed locally via PyPI, Bioconda, or from source (see the GitHub repository for details).

## Code availability

Project name: CADD-ThresholdApp. Project home page: [https://cadd-threshold.kircherlab.bihealth.org/]. Operating system(s): Platform independent. Programming language: Python. Other requirements: Python 3.10 or higher, shiny, plotly, scikit-learn. License: MIT License. Any restrictions to use by non-academics: No restrictions.

## Abbreviations

CADD: Combined annotation dependent depletion
CHROM: Chromosome
ClinVar: Clinical variation
GRCh37: Genome reference consortium human build 37
GRCh38: Genome reference consortium human build 38
POS: Position
REF: Reference allele
ALT: Alternate allele
PHRED: PHRED-scaled score
VCF: Variant call format
